# Vaccination coverage and timeliness in three South African areas: a prospective study

**DOI:** 10.1186/1471-2458-11-404

**Published:** 2011-05-27

**Authors:** Lars T Fadnes, Debra Jackson, Ingunn MS Engebretsen, Wanga Zembe, David Sanders, Halvor Sommerfelt, Thorkild Tylleskär

**Affiliations:** 1 Centre for International Health, University of Bergen, Norway; 2 School of Public Health, University of Western Cape, South Africa; 3 Medical Research Council, South Africa; 4 Division of Infectious Disease Control, Norwegian Institute of Public Health, Norway

## Abstract

**Background:**

Timely vaccination is important to induce adequate protective immunity. We measured vaccination timeliness and vaccination coverage in three geographical areas in South Africa.

**Methods:**

This study used vaccination information from a community-based cluster-randomized trial promoting exclusive breastfeeding in three South African sites (Paarl in the Western Cape Province, and Umlazi and Rietvlei in KwaZulu-Natal) between 2006 and 2008. Five interview visits were carried out between birth and up to 2 years of age (median follow-up time 18 months), and 1137 children were included in the analysis. We used Kaplan-Meier time-to-event analysis to describe vaccination coverage and timeliness in line with the Expanded Program on Immunization for the first eight vaccines. This included Bacillus Calmette-Guérin (BCG), four oral polio vaccines and 3 doses of the pentavalent vaccine which protects against diphtheria, pertussis, tetanus, hepatitis B and Haemophilus influenzae type B.

**Results:**

The proportion receiving all these eight recommended vaccines were 94% in Paarl (95% confidence interval [CI] 91-96), 62% in Rietvlei (95%CI 54-68) and 88% in Umlazi (95%CI 84-91). Slightly fewer children received all vaccines within the recommended time periods. The situation was worst for the last pentavalent- and oral polio vaccines. The hazard ratio for incomplete vaccination was 7.2 (95%CI 4.7-11) for Rietvlei compared to Paarl.

**Conclusions:**

There were large differences between the different South African sites in terms of vaccination coverage and timeliness, with the poorer areas of Rietvlei performing worse than the better-off areas in Paarl. The vaccination coverage was lower for the vaccines given at an older age. There is a need for continued efforts to improve vaccination coverage and timeliness, in particular in rural areas.

**Trial registration number:**

ClinicalTrials.gov: NCT00397150

## Background

Timely vaccination is important to induce adequate immunity [1-3]. Delayed immunisation is a risk factor for pertussis, measles and Haemophilus influenzae type B disease [1,2,4,5]. Late administration of the Bacillus Calmette-Guérin (BCG) vaccine is also associated with reduced survival [6].

Some studies have shown that good vaccination coverage for individual vaccines does not necessarily imply timely vaccination [3,7-11]. There may also be non-specific effects of vaccines that can be influenced by timeliness, with potential negative consequences of delayed immunisation [12,13]. Thus, it is important to take timeliness into account, as relying only on vaccination status can lead to a false assumption of adequate vaccination.

Although some studies have evaluated timely vaccination of some selected vaccines, we are only aware of two published studies from the United States and Uganda where the timeliness of all nationally recommended vaccines has been evaluated [10,11]. Only two other studies have assessed timely vaccination for some selected vaccines in African settings [9,14]. There also seems to be conflicting information in South Africa between the nationally reported morbidity related to vaccine preventable diseases compared to reports about large measles outbreaks [15,16]. In this study, we assessed immunisation timeliness and vaccination coverage for the first eight vaccines of the Expanded Program on Immunization (EPI) in South Africa. This included BCG (Bacillus Calmette-Guérin), four doses of oral polio vaccines and three doses of the pentavalent vaccine protecting against diphtheria, pertussis, tetanus, hepatitis B and Haemophilus influenzae type B disease.

## Methods

This study used vaccination information from the community-based cluster-randomized PROMISE-EBF trial promoting exclusive breastfeeding by peer-counsellors in three South African sites between 2006 and 2008 (ClinicalTrials.gov no: NCT00397150) [17]. A total of 34 clusters from three separate areas in South Africa were chosen: Paarl in Western Cape Province (peri-urban), and Umlazi (peri-urban) and Rietvlei (rural) in KwaZulu-Natal Province. The infant mortality rate (IMR) in Paarl is about 40/1000, and the antenatal HIV-prevalence was 10% at the time of the study [18]. The IMR in Umlazi was around 60/1000 and the antenatal HIV-prevalence 42%. The poor and rural area Rietvlei had an IMR of 99/1000 and an antenatal HIV-prevalence of 34% at the time of the study.

Some of the, mothers approached for study participation in the PROMISE-EBF study were excluded due to an intention to formula feed (142). They were enrolled in the Good start Study [19]. Both the participants in the PROMISE-EBF study and the Good Start Study provided information on vaccination. A total of 1276 mother-infant pairs were recruited from both of the studies. Among these, 139 were excluded due to relocation or being lost-to-follow-up, twin delivery, death of the infant or mother before 3 weeks after birth, or severe malformations (additional file 1, figure S1). Thus, 1137 mother-infant pairs remained in the analysis. The mother-infant pairs were scheduled to be interviewed at 3, 6, 12 and 24 weeks after birth, with an additional follow-up interview between 18 and 24 months of age. Most vaccination information was collected during the last two visits. Vaccination information was collected from the last two visits. A health card was seen for 935 (98%) among those who were interviewed at 24 weeks when much of the vaccination information was gathered. These health cards were dated, but in some instances the date was invalid. The median follow-up time was 18 months.

### Data management

Data was collected through interviews by trained data collectors, and subsequently entered using EpiData (http://www.epidata.dk). Data analysis was done with Stata version SE11.1 (Stata Corporation).

### Vaccination timeliness and coverage

The Expanded Program on Immunization in South Africa recommended the following vaccines to be given at specific ages during the study period in line with the recommendations from the World Health Organisation (time ranges given in parentheses) [9,20]:

○ The first vaccination visit is at birth when the BCG (birth-8 weeks) and oral polio (birth-4 weeks) vaccines are given.

○ The following three vaccination visits include the oral polio vaccine and a pentavalent vaccine which protects against diphtheria, tetanus and pertussis (DTP), *Haemophilus influenzae *type B (Hib) disease and hepatitis B (HBV). The first dose is given at 6 weeks (4 weeks-2 months), then again at 10 weeks (8 weeks-4 months), and at 14 weeks (12 weeks-6 months).

○ The measles vaccine is given at 9 months (38 weeks-12 months).

○ At 18 months, a booster dose was given with protection against measles, polio, diphtheria, pertussis and tetanus. The recommended time range of the booster vaccination has not been clearly defined.

○ In 2009 (after completed data collection), a rotavirus and a 7-valent pneumococcal vaccine were added to the program.

Vaccines given outside these ranges will be referred to as untimely vaccinations.

### Analysis

With the exceptions of measles and booster vaccines, vaccination timeliness was analysed with Kaplan-Meier time-to-event analysis in line with Laubereau *et al*. [21]. Coverage was determined at the end of follow-up. Vaccination data were gathered from the children's health cards. When vaccinations were registered without a valid date, we assumed that the age when the children were given the specific vaccines was similar to the age of those who had their vaccinations dated (around 2% had missing dates for each vaccine). The confidence intervals (CI) were estimated with Greenwood's pointwise method. The timing of measles and booster vaccines is presented with histograms. Due to a question that was missed out in the questionnaire, we had to make an assumption whether the reported timing of the measles vaccine was either the first measles vaccine or the booster vaccine. We assumed that vaccinations received before 12 months of age were the first dose of measles vaccine, and vaccinations after 12 months were booster vaccinations. Due to the structural weaknesses in the recording of measles vaccination in this study, the information on measles vaccination must be interpreted with care.

To investigate determinants of timely vaccination, we used a cluster adjusted Cox regression analysis. The Cox regression assumption of proportional hazards was checked with Schoenfeld residuals, both graphically and with a significance test. Tied cases were handled with the exact partial-likelihood method. Rational interactions were evaluated. Log linearity was checked by plotting of Martingale residuals for the complete model vs. a model with one omitted variable. We present univariable and multivariable models for both coverage at the end of follow-up and timeliness. The multivariable analyses used a stepwise selection with removal of covariates when p > 0.1. The models were adjusted for cluster.

Socioeconomic wealth index was constructed with the use of multiple correspondence analysis based on ownership of assets as mobile phone and television, and house characteristics including water source, roof material and toilet type. This method is analogous to principal component analysis, and better suited for categorical data [22]. The children's families were grouped into quintiles on the basis of socioeconomic rank.

### Ethics

Ethical approval was granted by Ethics Committee of the Medical Research Council South Africa, and Regional Committees for Medical and Health Research Ethics, Western Norway. Signed or thumb-printed informed consent was obtained from each mother prior to study participation.

## Results

There were large variations between the three sites in vaccination coverage and timeliness, both for the individual vaccines and all the first eight vaccines assessed together, table 1, figures 1, 2 and 3. The timing of the individual vaccines and the timing of all vaccines presented with hazard plot are available as additional files 2, 3, 4, 5, 6, 7, 8, 9 and10 (figures S2, S3, S4, S5, S6, S7, S8, S9 andS10).

**Table 1:** Coverage and timeliness of vaccines in the South African EPI program.

	Site	Coverage (end of follow-up)	Timely vaccination	Given too early	Given too late	Not given (at end of follow-up)
**Polio 0**	Paarl	99 (96-100)	99 (96-100)	0 (0-0)	0 (0-0)	1 (0-4)
	Umlazi	100 (98-100)	99 (97-100)	0 (0-0)	1 (0-1)	0 (0-2)
	Rietvlei	99 (96-100)	94 (91-97)	0 (0-0)	4 (3-6)	1 (0-4)
	**Total**	**100 (99-100)**	**97 (96-98)**	**0 (0-0)**	**3 (2-3)**	**0 (0-1)**

**BCG vaccine**	Paarl	99 (97-100)	99 (97-100)	0 (0-0)	0 (0-0)	1 (0-3)
	Umlazi	100 (98-100)	100 (98-100)	0 (0-0)	0 (0-0)	0 (0-2)
	Rietvlei	99 (97-100)	99 (97-100)	0 (0-0)	0 (0-0)	1 (0-3)
	**Total**	**99 (98-100)**	**99 (98-100)**	**0 (0-0)**	**0 (0-0)**	**1 (0-2)**

**Polio 1**	Paarl	100 (98-100)	92 (90-93)	1 (0-2)	7 (5-8)	0 (0-2)
	Umlazi	99 (98-100)	88 (86-89)	1 (0-3)	10 (8-12)	1 (0-2)
	Rietvlei	96 (92-98)	72 (67-75)	1 (0-4)	22 (19-25)	4 (2-8)
	**Total**	**99 (98-99)**	**85 (84-87)**	**1 (1-2)**	**12 (11-14)**	**1 (1-2)**

**DPT1+Hib+HBV**	Paarl	100 (98-100)	93 (90-94)	0 (0-2)	6 (4-8)	0 (0-2)
	Umlazi	99 (98-100)	88 (86-89)	1 (1-3)	10 (8-12)	1 (0-2)
	Rietvlei	97 (95-99)	77 (73-79)	2 (1-4)	19 (16-21)	3 (1-5)
	**Total**	**99 (98-100)**	**87 (85-88)**	**1 (1-2)**	**11 (10-12)**	**1 (0-2)**

**Polio 2**	Paarl	99 (97-100)	94 (92-94)	1 (0-2)	4 (3-5)	1 (0-3)
	Umlazi	98 (96-99)	90 (87-91)	0 (0-2)	8 (6-9)	2 (1-4)
	Rietvlei	87 (82-91)	72 (67-75)	1 (0-3)	14 (12-14)	13 (9-18)
	**Total**	**96 (94-97)**	**87 (85-88)**	**1 (0-1)**	**8 (7-9)**	**4 (3-6)**

**DPT2+Hib+HBV**	Paarl	99 (97-100)	95 (93-95)	0 (0-2)	3 (2-4)	1 (0-3)
	Umlazi	98 (96-99)	90 (88-91)	0 (0-2)	7 (6-8)	2 (1-4)
	Rietvlei	92 (88-95)	80 (75-82)	0 (0-3)	12 (10-13)	8 (5-12)
	**Total**	**97 (96-98)**	**90 (88-91)**	**0 (0-1)**	**7 (6-8)**	**3 (2-4)**

**Polio 3**	Paarl	94 (91-96)	93 (91-94)	1 (0-3)	0 (0-0)	6 (4-9)
	Umlazi	98 (96-99)	87 (83-88)	0 (0-2)	11 (9-13)	2 (1-4)
	Rietvlei	69 (62-75)	64 (57-71)	0 (0-0)	5 (4-5)	31 (25-38)
	**Total**	**87 (85-89)**	**84 (82-86)**	**0 (0-1)**	**2 (2-3)**	**13 (11-15)**

**DPT3+Hib+HBV**	Paarl	94 (91-96)	92 (90-93)	1 (0-3)	0 (0-0)	6 (4-9)
	Umlazi	90 (87-93)	86 (83-88)	0 (0-2)	4 (3-4)	10 (7-13)
	Rietvlei	74 (68-80)	70 (64-73)	0 (0-3)	4 (4-4)	26 (20-32)
	**Total**	**88 (86-90)**	**85 (83-86)**	**1 (0-1)**	**3 (3-3)**	**12 (10-14)**

Coverage at end of follow-up and the proportion receiving the vaccinations within the recommended time periods (timely vaccination) are presented with 95% confidence intervals. Similarly, untimely vaccination are categorised into vaccines given earlier or later than recommended. Rates given for each of the three sites and in total

* The pentavalent vaccine (DPT1+Hib+HBV) protects against diphtheria, pertussis, tetanus, Haemophilus influenzae type b and hepatitis B

**Figure 1:**
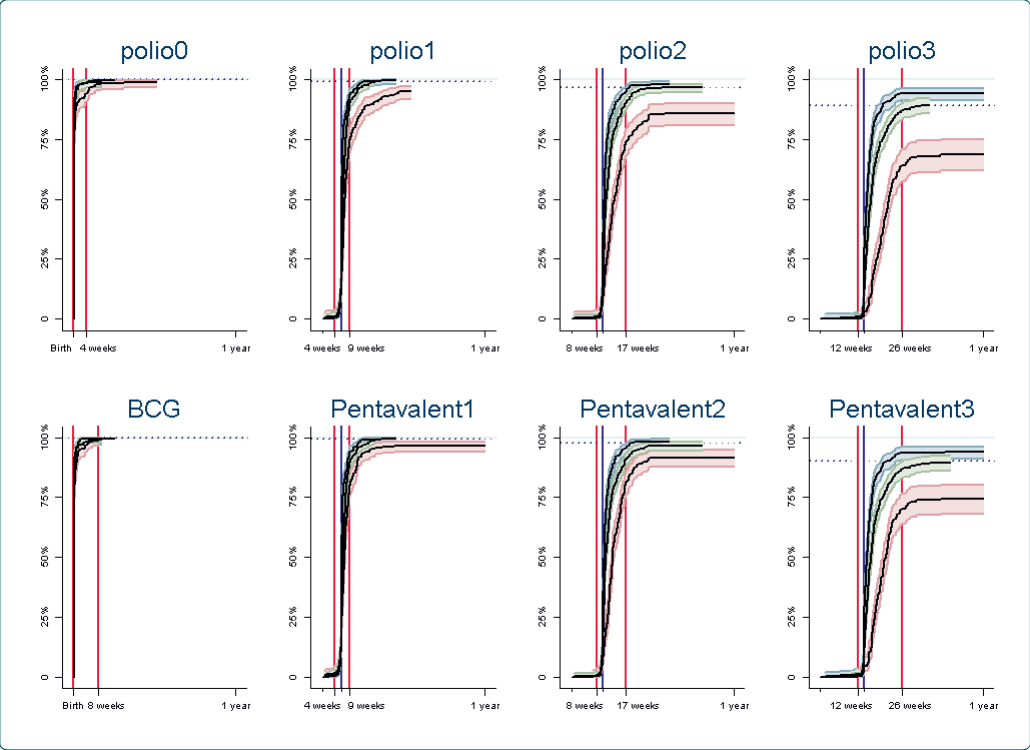
**Time when receiving vaccines given in the four first vaccination visits for each site presented with inverse and cumulative Kaplan-Meier plots **. In the first vaccination visit, an oral polio vaccine (polio0) is given together with the BCG vaccine, in the second, third and fourth vaccination visits, pentavalent vaccines are given together with polio vaccines. (Larger versions of the graph images are available as additional files 3, 4, 5, 6, 7, 8, 9 and10.) ^1 ^The blue vertical lines indicate the recommended age for vaccination (overlapping with red lines at birth for BCG and first polio vaccine), while the red lines indicate the outer ranges for the recommended age. The horizontal dotted lines represent coverage at end of follow-up. ^2 ^The labels on the x-axis indicate the outer ranges for recommended vaccination age. One year of age is indicated as a scaling, but is also the upper recommended age for the measles vaccine. ^3 ^Blue graphs line: Paarl; green graph line: Umlazi; red graph line: Rietvlei.

**Figure 2:**
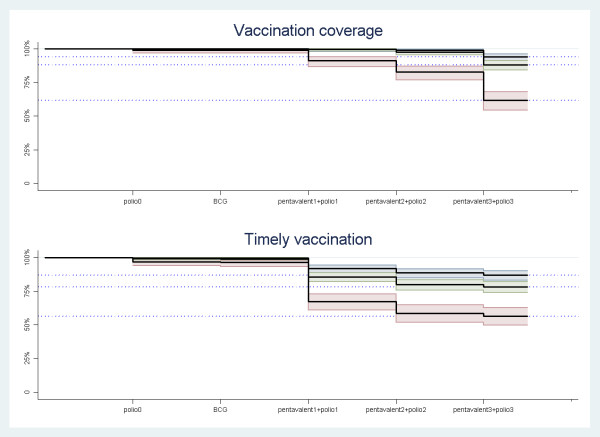
**Cumulative incomplete vaccination and untimely vaccination from birth to 6 months of age (including vaccination with BCG, 4 doses of oral polio vaccines, and 3 doses of pentavalent vaccines) **. ^1 ^Coverage: The line drops represent the proportion that has got all the vaccines to that point, but does not get the given vaccine (e.g. if a child get all vaccines except the last pentavalent vaccine, the child will add to the line drop at polio3/DTP3) ^2 ^Timeliness: The line drops represent the proportion that has got all the vaccines to that point within their recommended time ranges, but does not get the given vaccine within the recommended range (e.g. if a child get all vaccines within recommend time except the DPT3 vaccine, the child will add to the line drop at third pentavalent) ^3 ^Measles vaccine and booster vaccination not included due to sub-optimal data quality

**Figure 3:**
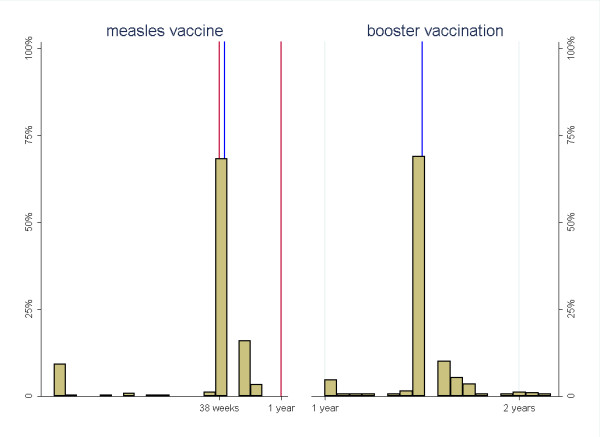
**Time when receiving the measles vaccine and booster vaccination with measles, DPT and oral polio presented with histogram (must be interpreted with caution) **. ^1 ^The blue vertical lines indicate the recommended age for vaccination, while the red lines on the measles vaccine indicate the outer ranges for the recommended age. ^2 ^When the booster vaccine against measles was given at different time from the booster against polio and DPT (3 cases), the time of the measles booster will be presented.

The proportion of children receiving all of the first eight recommended vaccines were 94% in Paarl (95%CI 91-96), 88% in Umlazi (95%CI 84-91) and 62% in Rietvlei (95%CI 54-68). Fewer children received all vaccines within the recommended time period, 88% in Paarl (95% confidence interval 84-91), 79% in Umlazi (95%CI 74-82) and 58% in Rietvlei (95%CI 51-64). Nearly all untimely vaccinations were given too late.

The coverage was lowest for the last oral polio vaccine with a coverage rate of 87% (95%CI 85-89) for all sites combined, and for the last pentavalent vaccine with coverage of 88% (95%CI 86-90). Rietvlei had the lowest coverage also for these vaccines.

Among the children above 12 months of age, 669 (86%) had received at least one measles vaccine. The time for the measles vaccination was distributed around the recommended time period of 38 weeks, figure 3. The distribution of booster vaccination was around 18 months with a small amount of scattering.

### Determinants of incomplete and untimely vaccination

The mean level of education among the mothers was 10 years of schooling in Paarl, 11 years in Umlazi and 9 years in Rietvlei. The socioeconomic situation was different in the 3 sites, reflected in 328 (94%) having electricity in Paarl, 446 (93%) in Umlazi, and 185 (61%) in Rietvlei.

No interactions were significant and meaningful in magnitude compared to the individual factors. No combination variables had correlation values above 0.7. The Cox regression models revealed a strong association between geographical site and both incomplete vaccinated at the end of follow-up and untimely vaccination, table 2. The hazard ratio (HR) for incomplete vaccinated was 7.2 (95%CI 4.7-11) for Rietvlei compared to Paarl. The association between geographical site and untimely vaccination was slightly weaker, with a HR 3.3 (95%CI 2.3-4.7) for Rietvlei compared to Paarl.

**Table 2:** Baseline characteristics of study participants presented with number (n) and percentages, determinants of untimely vaccination, and determinants of incomplete vaccination at end of follow-up presented with hazard ratios (HR) with 95% confidence interval assessed with a cluster adjusted Cox regression model.

	n (%) 765	HR for untimely Unadjusted model	vaccination (95%CI) Adjusted model	HR for incomplete Unadjusted model	vaccination (95%CI) Adjusted model
**Mother's age**					
≤ 19	265 (23)	1		1	
20 - 24	387 (34)	0.77 (0.57-1.0)		0.70 (0.47-1.1)	
25 - 29	249 (22)	0.91 (0.68-1.2)		0.59 (0.41-0.85)	
≥ 30	234 (21)	0.90 (0.65-1.2)		0.96 (0.66-1.4)	
					
**Marital status**		*		*	
Married or cohabiting	712 (63)	1		1	
Single, widowed, separated or divorced	422 (37)	0.64 (0.51-0.81)		0.65 (0.45-0.95)	
					
**Gender of infant**					
Girl	568 (50)	1	1	1	
Boy	559 (50)	1.3 (1.0-1.6)	1.2 (0.92-1.5)	0.95 (0.68-1.3)	
					
**Mother's education**				*	
Primary (0-7 years)	142 (11)	1		1	
Secondary (8-11 years)	756 (59)	1.2 (0.80-1.7)		0.62 (0.40-0.96)	
Higher (12 or above)	378 (30)	0.81 (0.53-1.2)		0.31 (0.19-0.53)	
					
**Socio economic wealth index**		*		*	
Least poor quintile	234 (21)	1		1	
4^th ^quintile	253 (22)	0.91 (0.56-1.5)		1.4 (0.70-3.0)	
3^rd ^quintile	223 (20)	1.1 (0.69-1.8)		2.2 (1.1-4.4)	
2^nd ^quintile	210 (18)	1.5 (1.0-2.4)		3.6 (1.8-7.0)	
Poorest quintile	217 (19)	2.4 (1.6-3.6)		7.5 (4.0-14)	
					
**Living area**		*	*	*	*
Paarl	349 (31)	1	1	1	1
Rietvlei	305 (27)	3.8 (2.7-5.5)	3.3 (2.3-4.7)	7.4 (4.4-13)	7.2 (4.7-11)
Umlazi	480 (42)	1.7 (1.3-2.4)	1.6 (1.2-2.3)	1.9 (1.1-3.5)	2.2 (1.3-3.7)
					
**Place of delivery**		*	*	*	
Home	68 (6)	1	1	1	
In health facility	1021 (94)	0.37 (0.26-0.54)	0.53 (0.38-0.74)	0.36 (0.20-0.64)	
					
**Health councelling**				*	*
Normal	485 (43)	1		1	1
Additional peer-counselling	535 (47)	0.88 (0.56-1.4)		0.67 (0.35-1.28)	0.64 (0.49-0.83)
Intention to formula feed	117 (10)	0.74 (0.41-1.3)		0.40 (0.16-0.96)	0.39 (0.18-0.89)
					
**Number of siblings**				*	*
None	537 (47)	1		1	1
1 - 2	464 (41)	1.0 (0.84-1.3)		1.3 (1.04-1.7)	1.3 (0.97-1.7)
3 or above	130 (11)	1.4 (1.08-1.9)		2.2 (1.4-3.5)	1.7 (1.0-2.7)
					
**Mother's body mass index (BMI)**					
< 20	70 (8)	1		1	
20 - 24.9	361 (39)	1.3 (0.76-2.1)		0.82 (0.44-1.5)	
25 - 29.9	283 (31)	1.2 (0.69-2.2)		0.77 (0.40-1.5)	
≥ 30	211 (23)	1.5 (0.88-2.5)		0.76 (0.43-1.3)	

* Significantly associated variables (both categorised and socio-economy also uncategorised)

Interactions were checked and none were considered meaningful to include

All factors are cluster adjusted

The children being delivered at home or children with several siblings also had a higher risk of incomplete and untimely vaccination. Socioeconomic factors were also strongly associated with timely vaccination and vaccination coverage in the univariable analysis, but was nearly nullified when including geographical site in the model. A site stratified analysis confirmed that socio-economy within the site was not associated with vaccination coverage in Rietvlei and Umlazi, but an association was seen in Paarl (data not shown).

Breastfeeding peer-counselling was also associated with vaccination coverage, with higher coverage among children receiving peer-counselling on breastfeeding and children of mothers who intended to formula feed.

## Discussion

This study revealed substantial differences in vaccination between the different geographical sites. There was a 7 fold hazard ratio of not being vaccinated at the end of follow-up for Rietvlei compared to Paarl. Rietvlei is a poor and rural area, but adjusting for socio economic or other factors did not alter the association substantially. This is consistent with other studies in these three populations which have shown strong geographic differences in health services quality and health outcomes [19].

The nationally reported coverage rates for the third doses of the HBV and Hib vaccines in 2004 were 92% and 93%, respectively [23]. This is somewhere between the estimates for Paarl and Umlazi that is reported in this study for the third dose of the corresponding pentavalent vaccine in this study, but much higher than the coverage rates in the poor area Rietvlei.

The differences between vaccination coverage and vaccination timeliness were not as large for most vaccines in this study compared to what has been observed in some other studies [3,7-10]. This means that most of those who were vaccinated, received their vaccines within the recommended time period. Still, the proportion who received their vaccines later than recommended for the second and third vaccination visits was substantial. In general, the factors associated with good vaccination coverage were the factors that were associated with timely vaccination.

Although it is difficult to assess the timeliness and coverage of measles vaccination from this study, the trend with increasingly lower coverage for the later vaccinations compared to the earlier vaccinations, indicate that the situation for the measles vaccine is likely to be worse than for the last pentavalent and oral polio vaccines. Currently, the South African vaccination program has included the rotavirus and pneumococcal vaccines, which have a potential to avert a large number of child deaths [24]. These vaccines are supposed to be given together with the pentavalent and measles vaccines. Thus, sub-optimal vaccination with for example the third pentavalent vaccine is likely to be associated with unsatisfactory vaccination also with the rotavirus and pneumococcal vaccines. To get optimal protection from these vaccines, good vaccination coverage and timeliness are essential [25,26].

Globally there have been gradual improvements in vaccination coverage, with large reductions in measles, pertussis and tetanus mortality. Still, these diseases were responsible for about 4% of the global child mortality according to a systematic analysis from 2008 [15]. The same report attributed few deaths in South Africa to these diseases, with only one pertussis death, one measles death, and 139 deaths from tetanus. This is likely to be an underestimate, considering the relatively low coverage rates we observed in Rietvlei for the vaccines given earlier than the measles vaccine, in addition to large measles outbreaks in South Africa, including an extensive national outbreak reported in June 2010 [16]. More than 15 000 cases with measles and 18 confirmed deaths were report from this source.

Most deaths from diseases with available vaccines are preventable, and diseases such as measles and polio can be eliminated with vaccination [27-29]. To accomplish this, a high vaccination coverage rate is needed [30,31]. The vaccination coverage in this study was inadequate particularly for the later vaccines in some of the areas, and several children were also vaccinated too late, leaving them susceptible to diseases when their maternal antibodies have declined to levels insufficient to protect them [1-3]. For some vaccines including the BCG vaccine, late administration has been associated with higher mortality [6]. In this study, nearly all received the BCG and first oral polio vaccine soon after birth. There is also a lack of evidence on potential indirect effects of timing of immunisation outside the recommended time ranges [12].

The presence of the measles booster vaccine in the South African immunisation program offers some insurance if children are either vaccinated earlier than recommended or miss the first measles vaccine [30,32-34]. One of the associations that was found in this study was a lower risk of incomplete vaccination among those who had an intention to formula feed. This might be a reflection that many of these children have HIV-positive mothers, and they might be followed up more closely, also with respect to vaccination of their children. For children infected with HIV, the decision on when to vaccinate is further complicated as severe immune suppression is a contraindication to vaccination with live vaccines such as the measles vaccine [34]. However, those with symptomatic HIV infection but who are not severely immuno compromised are considered for vaccination [20].

There was also an association with lower risk of incomplete immunisation among children receiving breastfeeding counselling than children in the control arm. Despite the fact that this intervention did not prove particularly effective in increasing the practice of exclusive breastfeeding in South Africa (submitted) and even though the mothers were not counselled on vaccines, there was a general focus on child health in addition to the breastfeeding in the intervention arm of the study which might explain the positive impact on vaccination.

To have a good vaccination program, a well-functioning health system is required [35], where both individual and contextual factors are of importance for utilization of the health services [36]. The very substantial differences in vaccination coverage between the three sites in this study are largely a reflection of the relative strengths and functionality of the health systems in these three areas, as well as the large differences in socio economic status [37]. Achieving good and timely vaccination coverage and eliminating the vast disparities between the different geographical areas will require both general developmental interventions - concerning education, infrastructure and communications - as well as health system strengthening. The latter efforts will need to focus on good coverage by health services, particularly at community and primary levels with effective referral mechanisms, well-equipped and organised clinics with short waiting times and effective integration of maternal and child health activities, as well as good media promotion and campaigns [35,38]. Vaccination reminders are an effective strategy to improve vaccination, particularly phone call reminders [39]. The feasibility of instituting vaccination reminders should be explored, given that possession and use of cell phones in South Africa has increased dramatically in the recent past.

This study has some limitations. The time estimate of the first measles vaccination is likely to be biased to vaccination before 12 months because a question from the last interview was missed out. Thus, the time distribution of the measles vaccination must be interpreted with caution. Some of the last follow-up interviews were also done too early to assess the booster vaccination, but this is likely to have affected the estimates less than for the first measles vaccine.

The children who died during follow-up might have had different vaccination status compared to the surviving majority [40], but mortality was low and therefore this is unlikely to have biased our estimates. Most of the vaccination information was collected in the 24 weeks interview and those who were not interviewed at that point and did not provide sufficient vaccination information in the other interviews visits might also have had a different vaccination status. The proportion of participants with insufficient information to be assessed was 13%. These participants were more often from the poorer areas, and thus the combined estimates might be slightly lower than what has been presented in this paper. The timeliness of vaccination in the few children who had immunisation indicated as received but not dated in the health card could be different from the majority where it was dated. As there were only a few and that they had similar baseline characteristics (data not shown), we believe this has not biased our estimates markedly. Contraindications for vaccination were not assessed [41], but this is relevant only in a few cases. It may be justified to temporarily postpone vaccination in some few cases when children are moderately or severely ill. Some children may have been HIV-positive with severe immune suppression, which is a contraindication for live vaccines such as the measles vaccine and complicates the assessment of coverage and timeliness [34,41]]. In this study, many of the mothers in the intention to formula feed arm were HIV-positive, but the number of infected children is likely to be much less. As the Cox regression model assessed timeliness with specific accepted time ranges, there were several ties with equal time-to-event. To improve model robustness, we used the exact partial-likelihood method for handling ties. The study also has several strengths compared to many studies assessing vaccination; these include a prospective design, date assessment of the vaccines, assessment of all recommended vaccines, inclusion of different sites, and the use of time-to-event analysis.

## Conclusion

There are substantial differences in vaccination coverage and timely vaccination between different geographical areas in South Africa. There was a trend towards worse vaccination coverage with the later vaccines in the vaccination programme. To have adequate disease protection and to aim for elimination of some of the diseases, there is a need for continued efforts to improve vaccination coverage and timeliness.

There is also a need for efforts and research to assess whether general socio economic development as well as strengthening of the health systems could improve vaccination coverage and reduce disparities between different geographical areas and socio economic groups.

## Abbreviations

BCG: Bacillus Calmette-Guérin; CI: confidence interval; DTP: diphtheria, tetanus and pertussis; EPI: Expanded Programme on Immunization; HR: hazard ratio; HBV: hepatitis B; HiB: Haemophilus influenza type B; WHO: World Health Organisation.

## Competing interests

The authors declare that they have no competing interests.

## Authors' contributions

LTF: design, analysis and writing. DJ, WZ, DS: design, implementation and co-writing. IMSE, HS, TT: design, analysis and co-writing. The PROMISE-EBF Study Group: design and implementation. All authors read and approved the final manuscript.

## Pre-publication history

The pre-publication history for this paper can be accessed here:

http://www.biomedcentral.com/1471-2458/11/404/prepub

## Supplementary Material

Additional file 1 **Figure S1: Study profile**. Number of participants in the different trial arms: Good Start Study (intention to formula feed) and PROMISE-EBF study (intention to breastfeed) with its intervention and control arms. The number of participants interviewed in the different visits is given with proportion of the included participants in the parenthesis. Cumulative proportion of lost-to-follow-up is also added in brackets. Click here for file

Additional file 2 **Figure S2: Figure presenting age distribution when the different vaccines are given for all the sites combined (hazard plot). **^1 ^The blue vertical lines indicate the recommended age for vaccination (overlapping with red lines at birth for BCG and first polio vaccine), while the red lines indicate the outer ranges for the recommended age. The horizontal dotted lines represent coverage at end of follow-up. ^2 ^The labels on the x-axis indicate the outer ranges for recommended vaccination age. One year of age is indicated as a scaling, but is also the upper recommended age for the measles vaccine. Click here for file

Additional file 3 **Figure S3: Timing of the oral polio vaccine (polio0) given at birth for each site presented with Kaplan-Meier plots (inverse and cumulative). **^1 ^The blue vertical lines indicate the recommended age for vaccination (overlapping with red lines at birth for BCG and first polio vaccine), while the red lines indicate the outer ranges for the recommended age. The horizontal dotted lines represent coverage at end of follow-up. ^2 ^The labels on the x-axis indicate the outer ranges for recommended vaccination age. One year of age is indicated as a scaling, but is also the upper recommended age for the measles vaccine. ^3 ^Blue graphs line: Paarl; green graph line: Umlazi; red graph line: Rietvlei. Click here for file

Additional file 4 **Figure S4: Timing of the BCG vaccine given at birth for each site presented with Kaplan-Meier plots (inverse and cumulative). **^1 ^The blue vertical lines indicate the recommended age for vaccination (overlapping with red lines at birth for BCG and first polio vaccine), while the red lines indicate the outer ranges for the recommended age. The horizontal dotted lines represent coverage at end of follow-up. ^2 ^The labels on the x-axis indicate the outer ranges for recommended vaccination age. One year of age is indicated as a scaling, but is also the upper recommended age for the measles vaccine. ^3 ^Blue graphs line: Paarl; green graph line: Umlazi; red graph line: Rietvlei. Click here for file

Additional file 5 **Figure S5: Timing of the second oral polio vaccine (polio1) for each site presented with Kaplan-Meier plots (inverse and cumulative). **^1 ^The blue vertical lines indicate the recommended age for vaccination (overlapping with red lines at birth for BCG and first polio vaccine), while the red lines indicate the outer ranges for the recommended age. The horizontal dotted lines represent coverage at end of follow-up. ^2 ^The labels on the x-axis indicate the outer ranges for recommended vaccination age. One year of age is indicated as a scaling, but is also the upper recommended age for the measles vaccine. ^3 ^Blue graphs line: Paarl; green graph line: Umlazi; red graph line: Rietvlei. Click here for file

Additional file 6 **Figure S6: Timing of the first pentavalent vaccine (pentavalent1) for each site presented with Kaplan-Meier plots (inverse and cumulative). **^1 ^The blue vertical lines indicate the recommended age for vaccination (overlapping with red lines at birth for BCG and first polio vaccine), while the red lines indicate the outer ranges for the recommended age. The horizontal dotted lines represent coverage at end of follow-up. ^2 ^The labels on the x-axis indicate the outer ranges for recommended vaccination age. One year of age is indicated as a scaling, but is also the upper recommended age for the measles vaccine. ^3 ^Blue graphs line: Paarl; green graph line: Umlazi; red graph line: Rietvlei. Click here for file

Additional file 7 **Figure S7: Timing of the third oral polio vaccine (polio2) for each site presented with Kaplan-Meier plots (inverse and cumulative). **^1 ^The blue vertical lines indicate the recommended age for vaccination (overlapping with red lines at birth for BCG and first polio vaccine), while the red lines indicate the outer ranges for the recommended age. The horizontal dotted lines represent coverage at end of follow-up. ^2 ^The labels on the x-axis indicate the outer ranges for recommended vaccination age. One year of age is indicated as a scaling, but is also the upper recommended age for the measles vaccine. ^3 ^Blue graphs line: Paarl; green graph line: Umlazi; red graph line: Rietvlei. Click here for file

Additional file 8 **Figure S8: Timing of the second pentavalent vaccine (pentavalent2) for each site presented with Kaplan-Meier plots (inverse and cumulative). **^1 ^The blue vertical lines indicate the recommended age for vaccination (overlapping with red lines at birth for BCG and first polio vaccine), while the red lines indicate the outer ranges for the recommended age. The horizontal dotted lines represent coverage at end of follow-up. ^2 ^The labels on the x-axis indicate the outer ranges for recommended vaccination age. One year of age is indicated as a scaling, but is also the upper recommended age for the measles vaccine. ^3 ^Blue graphs line: Paarl; green graph line: Umlazi; red graph line: Rietvlei. Click here for file

Additional file 9 **Figure S9: Timing of the fourth oral polio vaccine (polio3) for each site presented with Kaplan-Meier plots (inverse and cumulative). **^1 ^The blue vertical lines indicate the recommended age for vaccination (overlapping with red lines at birth for BCG and first polio vaccine), while the red lines indicate the outer ranges for the recommended age. The horizontal dotted lines represent coverage at end of follow-up. ^2 ^The labels on the x-axis indicate the outer ranges for recommended vaccination age. One year of age is indicated as a scaling, but is also the upper recommended age for the measles vaccine. ^3 ^Blue graphs line: Paarl; green graph line: Umlazi; red graph line: Rietvlei. Click here for file

Additional file 10 **Figure S10: Timing of the third pentavalent vaccine (pentavalent3) for each site presented with Kaplan-Meier plots (inverse and cumulative). **^1 ^The blue vertical lines indicate the recommended age for vaccination (overlapping with red lines at birth for BCG and first polio vaccine), while the red lines indicate the outer ranges for the recommended age. The horizontal dotted lines represent coverage at end of follow-up. ^2 ^The labels on the x-axis indicate the outer ranges for recommended vaccination age. One year of age is indicated as a scaling, but is also the upper recommended age for the measles vaccine. ^3 ^Blue graphs line: Paarl; green graph line: Umlazi; red graph line: Rietvlei. Click here for file

## References

[B1] Grant CC Roberts M Scragg R Stewart J Lennon D Kivell D Ford R Menzies R Delayed immunisation and risk of pertussis in infants: unmatched case-control study Brit Med J 2003 326 7394 852 853 10.1136/bmj.326.7394.852 12702617 PMC153471

[B2] Kolos V Menzies R McIntyre P Higher pertussis hospitalization rates in indigenous Australian infants, and delayed vaccination Vaccine 2007 25 4 588 590 10.1016/j.vaccine.2006.08.022 16971026

[B3] Heininger U Zuberbuhler M Immunization rates and timely administration in pre-school and school-aged children Eur J Pediatr 2006 165 2 124 129 10.1007/s00431-005-0014-y 16328365

[B4] von Kries R Bohm O Windfuhr A Haemophilus influenzae b-vaccination: the urgency for timely vaccination Eur J Pediatr 1997 156 4 282 287 10.1007/s004310050601 9128812

[B5] The measles epidemic. The problems, barriers, and recommendations. The National Vaccine Advisory Committee JAMA 1991 266 11 1547 1552 1880887

[B6] Breiman RF Streatfield PK Phelan M Shifa N Rashid M Yunus M Effect of infant immunisation on childhood mortality in rural Bangladesh: analysis of health and demographic surveillance data Lancet 2004 364 9452 2204 2211 10.1016/S0140-6736(04)17593-4 15610807

[B7] Akmatov MK Kretzschmar M Kramer A Mikolajczyk RT Timeliness of vaccination and its effects on fraction of vaccinated population Vaccine 2008 26 31 3805 3811 10.1016/j.vaccine.2008.05.031 18565626

[B8] Dayan GH Shaw KM Baughman AL Orellana LC Forlenza R Ellis A Chaui J Kaplan S Strebel P Assessment of delay in age-appropriate vaccination using survival analysis Am J Epidemiol 2006 163 6 561 570 10.1093/aje/kwj074 16421238

[B9] Clark A Sanderson C Timing of children's vaccinations in 45 low-income and middle-income countries: an analysis of survey data Lancet 2009 373 9674 1543 1549 10.1016/S0140-6736(09)60317-2 19303633

[B10] Luman ET Barker LE Shaw KM McCauley MM Buehler JW Pickering LK Timeliness of childhood vaccinations in the United States - Days undervaccinated and number of vaccines delayed Jama-J Am Med Assoc 2005 293 10 1204 1211 10.1001/jama.293.10.1204 15755943

[B11] Fadnes LT Nankabirwa V Sommerfelt H Tylleskar T Tumwine JK Engebretsen IM Is vaccination coverage a good indicator of age-appropriate vaccination? A prospective study from Uganda Vaccine 2011 29 19 3564 3570 10.1016/j.vaccine.2011.02.093 21402043

[B12] Shann F Nohynek H Scott JA Hesseling A Flanagan KL Randomized trials to study the nonspecific effects of vaccines in children in low-income countries Pediatr Infect Dis J 2010 29 5 457 461 10.1097/INF.0b013e3181c91361 20431383

[B13] Aaby P Rodrigues A Biai S Martins C Veirum JE Benn CS Jensen H Oral polio vaccination and low case fatality at the paediatric ward in Bissau, Guinea-Bissau Vaccine 2004 22 23-24 3014 3017 10.1016/j.vaccine.2004.02.009 15297050

[B14] Ndiritu M Cowgill KD Ismail A Chiphatsi S Kamau T Fegan G Feikin DR Newton CR Scott JA Immunization coverage and risk factors for failure to immunize within the Expanded Programme on Immunization in Kenya after introduction of new Haemophilus influenzae type b and hepatitis b virus antigens BMC Public Health 2006 6 132 10.1186/1471-2458-6-132 PMC1475578 16707013

[B15] Black RE Cousens S Johnson HL Lawn JE Rudan I Bassani DG Jha P Campbell H Walker CF Cibulskis R Global, regional, and national causes of child mortality in 2008: a systematic analysis Lancet 2010 375 9730 1969 1987 10.1016/S0140-6736(10)60549-1 20466419

[B16] WHO and UNICEF concerned about measles outbreak in Eastern and Southern Africa http://www.unicef.org/media/media_54018.html

[B17] Nankunda J Tylleskar T Ndeezi G Semiyaga N Tumwine JK Establishing individual peer counselling for exclusive breastfeeding in Uganda: implications for scaling-up Matern Child Nutr 2010 6 1 53 66 10.1111/j.1740-8709.2009.00187.x 20055930 PMC6860637

[B18] National Department of Health South Africa The national HIV and syphilis prevalence survey South Africa 2007 Pretoria, South Africa: National Department of Health 2007

[B19] Jackson DJ Chopra M Doherty TM Colvin MS Levin JB Willumsen JF Goga AE Moodley P Operational effectiveness and 36 week HIV-free survival in the South African programme to prevent mother-to-child transmission of HIV-1 AIDS 2007 21 4 509 516 10.1097/QAD.0b013e32801424d2 17301570

[B20] The South African EPI (EPI-SA) schedule and method of vaccine administration http://www.savic.ac.za

[B21] Laubereau B Hermann M Schmitt HJ Weil J von Kries R Detection of delayed vaccinations: a new approach to visualize vaccine uptake Epidemiol Infect 2002 128 2 185 192 12002536 10.1017/s0950268801006550 PMC2869811

[B22] Howe LD Hargreaves JR Huttly SR Issues in the construction of wealth indices for the measurement of socio-economic position in low-income countries Emerg Themes Epidemiol 2008 5 3 10.1186/1742-7622-5-3 18234082 PMC2248177

[B23] Arevshatian L Clements C Lwanga S Misore A Ndumbe P Seward J Taylor P An evaluation of infant immunization in Africa: is a transformation in progress? Bull World Health Organ 2007 85 6 449 457 10.2471/BLT.06.031526 17639242 PMC2636339

[B24] World Health Organization State of the world's vaccines and immunization 2009 3 http://www.who.int/immunization/sowvi/en/

[B25] Smith PJ Nuorti JP Singleton JA Zhao Z Wolter KM Effect of vaccine shortages on timeliness of pneumococcal conjugate vaccination: results from the 2001-2005 National Immunization Survey Pediatrics 2007 120 5 e1165 1173 10.1542/peds.2007-0037 17974712

[B26] Daskalaki I Spain CV Long SS Watson B Implementation of rotavirus immunization in Philadelphia, Pennsylvania: high levels of vaccine ineligibility and off-label use Pediatrics 2008 122 1 e33 38 10.1542/peds.2007-2464 18595974

[B27] Biellik R Madema S Taole A Kutsulukuta A Allies E Eggers R Ngcobo N Nxumalo M Shearley A Mabuzane E First 5 years of measles elimination in southern Africa: 1996-2000 Lancet 2002 359 9317 1564 1568 10.1016/S0140-6736(02)08517-3 12047966

[B28] Sudfeld CR Navar AM Halsey NA Effectiveness of measles vaccination and vitamin A treatment Int J Epidemiol 2010 39 Suppl 1 i48 55 20348126 10.1093/ije/dyq021 PMC2845860

[B29] Polio vaccines and polio immunization in the pre-eradication era: WHO position paper Wkly Epidemiol Rec 2010 85 23 213 228 20545051

[B30] Orenstein WA Strebel PM Papania M Sutter RW Bellini WJ Cochi SL Measles eradication: is it in our future? Am J Public Health 2000 90 10 1521 1525 10.2105/AJPH.90.10.1521 11029981 PMC1446359

[B31] Stittelaar KJ de Swart RL Osterhaus AD Vaccination against measles: a neverending story Expert Rev Vaccines 2002 1 2 151 159 10.1586/14760584.1.2.151 12901554

[B32] Dai B Chen ZH Liu QC Wu T Guo CY Wang XZ Fang HH Xiang YZ Duration of immunity following immunization with live measles vaccine: 15 years of observation in Zhejiang Province, China Bull World Health Organ 1991 69 4 415 423 1934235 PMC2393239

[B33] Strebel P Cochi S Grabowsky M Bilous J Hersh BS Okwo-Bele JM Hoekstra E Wright P Katz S The unfinished measles immunization agenda J Infect Dis 2003 187 Suppl 1 S1 7 12721885 10.1086/368226

[B34] World Health Organization Measles Vaccines: WHO Position Paper Weekly epidemiological record 2009 84 349 36019714924

[B35] Rwashana AS Williams DW Neema S System dynamics approach to immunization healthcare issues in developing countries: a case study of Uganda Health Informatics J 2009 15 2 95 107 10.1177/1460458209102971 19474223

[B36] Andersen RM National health surveys and the Behavioral Model of Health Services Use Med Care 2008 46 7 647 653 10.1097/MLR.0b013e31817a835d 18580382

[B37] Coovadia H Jewkes R Barron P Sanders D McIntyre D The health and health system of South Africa: historical roots of current public health challenges Lancet 2009 374 9692 817 834 10.1016/S0140-6736(09)60951-X 19709728

[B38] Ndiritu M Cowgill KD Ismail A Chiphatsi S Kamau T Fegan G Feikin DR Newton CRJC Scott JAG Immunization coverage and risk factors for failure to immunize within the Expanded Programme on Immunization in Kenya after introduction of new Haemophilus influenzae type b and hepatitis b virus antigens BMC Public Health 2006 6 10.1186/1471-2458-6-132 PMC1475578 16707013

[B39] Jacobson VJ Szilagyi P Patient reminder and patient recall systems to improve immunization rates Cochrane Database Syst Rev 2005 CD003941 3 10.1002/14651858.CD003941.pub2 PMC6485483 16034918

[B40] Jensen H Benn CS Lisse IM Rodrigues A Andersen PK Aaby P Survival bias in observational studies of the impact of routine immunizations on childhood survival Trop Med Int Health 2007 12 1 5 14 17207143 10.1111/j.1365-3156.2006.01773.x

[B41] Kroger AT Atkinson WL Marcuse EK Pickering LK General recommendations on immunization: recommendations of the Advisory Committee on Immunization Practices (ACIP) MMWR Recomm Rep 2006 55 RR-15 1 48 17136024

