# Supplementary material for: Vaccination coverage and timeliness in three South African areas: a prospective study
Source: BMC Public Health. 2011 May 27;11:404. doi: 10.1186/1471-2458-11-404 (PMC3126743; doi:10.1186/1471-2458-11-404)
Supplement: Additional file 1 — Figure S1: Study profile. Number of participants in the different trial arms: Good Start Study (intention to formula feed) and PROMISE-EBF study (intention to breastfeed) with its intervention and control arms. The number of participants interviewed in the different visits is given with proportion of the included participants in the parenthesis. Cumulative proportion of lost-to-follow-up is also added in brackets. [file 1471-2458-11-404-S1.PDF]

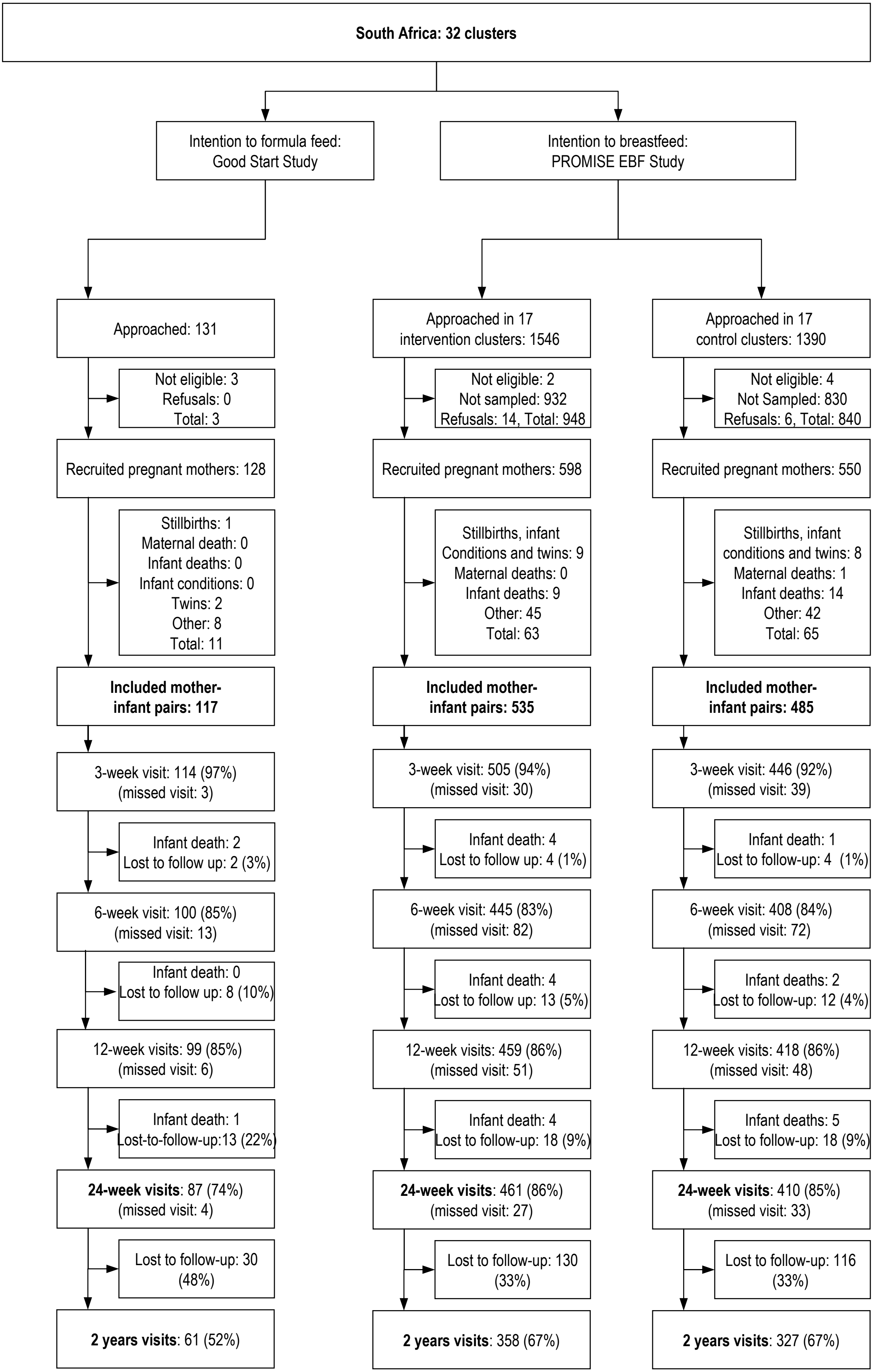
South Africa: 32 clusters
Intention to formula feed: Good Start Study
Intention to breastfeed: PROMISE EBF Study
Approached: 131
Not eligible: 3
Refusals: 0
Total: 3
Recruited pregnant mothers: 128
Stillbirths: 1
Maternal death: 0
Infant deaths: 0
Infant conditions: 0
Twins: 2
Other: 8
Total: 11
Included mother-infant pairs: 117
3-week visit: 114 (97%) (missed visit: 3)
Infant death: 2
Lost to follow up: 2 (3%)
6-week visit: 100 (85%) (missed visit: 13)
Infant death: 0
Lost to follow up: 8 (10%)
12-week visits: 99 (85%) (missed visit: 6)
Infant death: 1
Lost-to-follow-up:13 (22%)
24-week visits: 87 (74%) (missed visit: 4)
Lost to follow-up: 30 (48%)
2 years visits: 61 (52%)
Approached in 17 intervention clusters: 1546
Not eligible: 2
Not sampled: 932
Refusals: 14, Total: 948
Recruited pregnant mothers: 598
Stillbirths, infant Conditions and twins: 9
Maternal deaths: 0
Infant deaths: 9
Other: 45
Total: 63
Included mother-infant pairs: 535
3-week visit: 505 (94%) (missed visit: 30)
Infant death: 4
Lost to follow up: 4 (1%)
6-week visit: 445 (83%) (missed visit: 82)
Infant death: 4
Lost to follow up: 13 (5%)
12-week visits: 459 (86%) (missed visit: 51)
Infant death: 4
Lost to follow-up: 18 (9%)
24-week visits: 461 (86%) (missed visit: 27)
Lost to follow-up: 130 (33%)
2 years visits: 358 (67%)
Approached in 17 control clusters: 1390
Not eligible: 4
Not Sampled: 830
Refusals: 6, Total: 840
Recruited pregnant mothers: 550
Stillbirths, infant conditions and twins: 8
Maternal deaths: 1
Infant deaths: 14
Other: 42
Total: 65
Included mother-infant pairs: 485
3-week visit: 446 (92%) (missed visit: 39)
Infant death: 1
Lost to follow-up: 4 (1%)
6-week visit: 408 (84%) (missed visit: 72)
Infant deaths: 2
Lost to follow-up: 12 (4%)
12-week visits: 418 (86%) (missed visit: 48)
Infant deaths: 5
Lost to follow-up: 18 (9%)
24-week visits: 410 (85%) (missed visit: 33)
Lost to follow-up: 116 (33%)
2 years visits: 327 (67%)
