# Supplementary figures and images for: Vaccination coverage and timeliness in three South African areas: a prospective study
Source: BMC Public Health. 2011 May 27;11:404. doi: 10.1186/1471-2458-11-404 (PMC3126743; doi:10.1186/1471-2458-11-404)

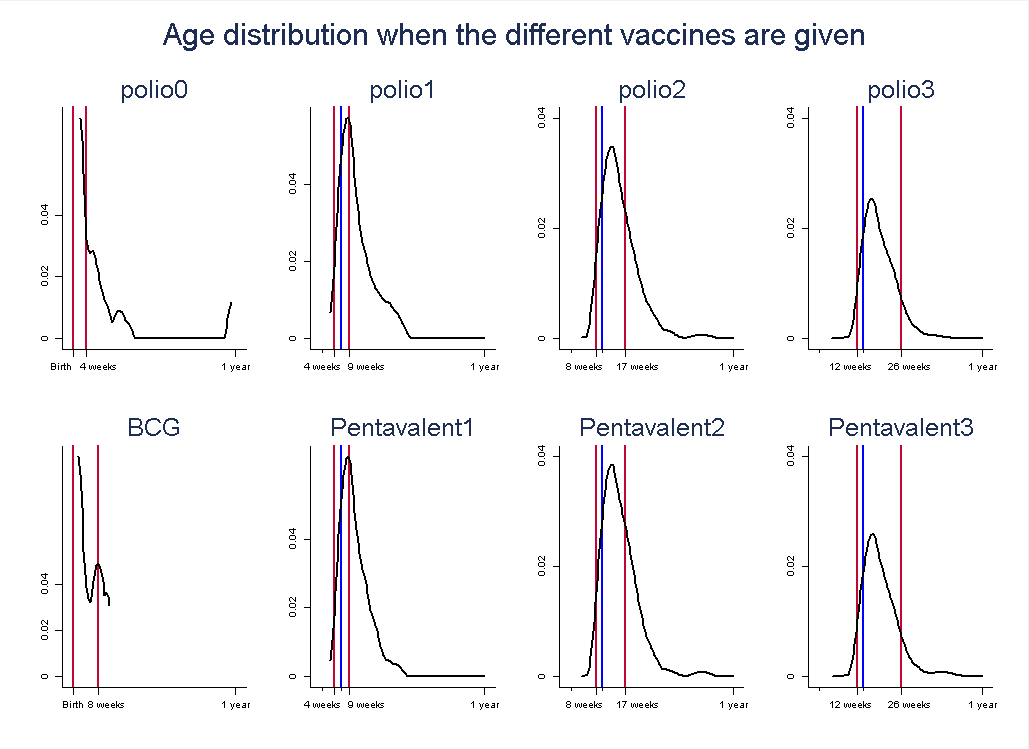

Supplement: Additional file 2 — Figure S2: Figure presenting age distribution when the different vaccines are given for all the sites combined (hazard plot). 1 The blue vertical lines indicate the recommended age for vaccination (overlapping with red lines at birth for BCG and first polio vaccine), while the red lines indicate the outer ranges for the recommended age. The horizontal dotted lines represent coverage at end of follow-up. 2 The labels on the x-axis indicate the outer ranges for recommended vaccination age. One year of age is indicated as a scaling, but is also the upper recommended age for the measles vaccine. [file 1471-2458-11-404-S2.TIFF]

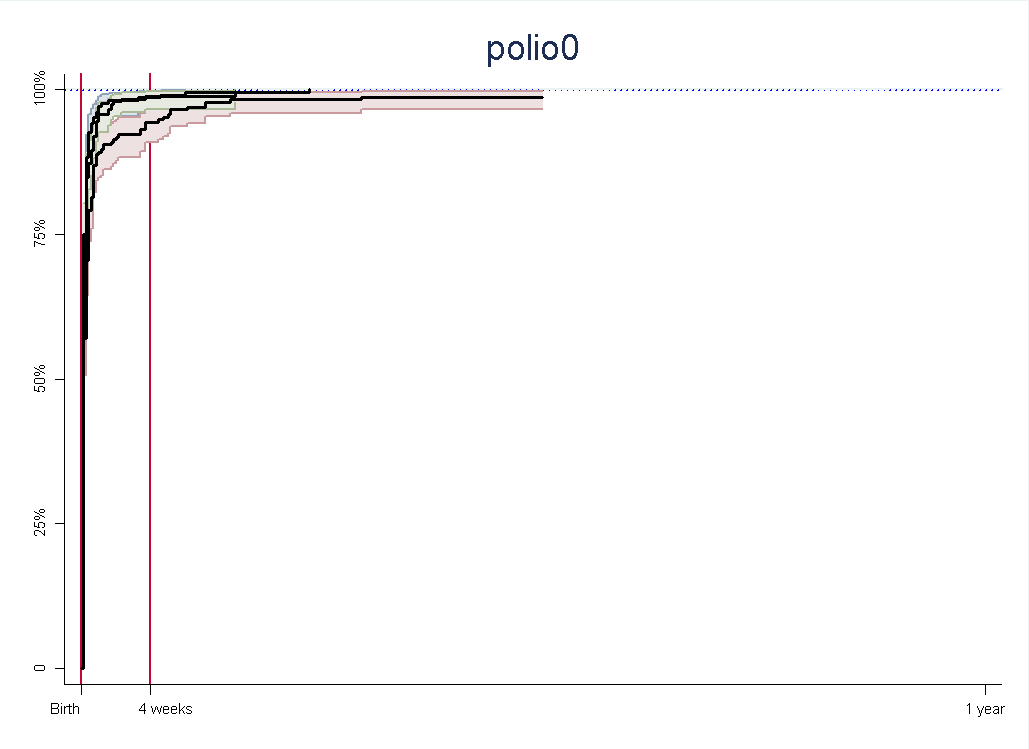

Supplement: Additional file 3 — Figure S3: Timing of the oral polio vaccine (polio0) given at birth for each site presented with Kaplan-Meier plots (inverse and cumulative). 1 The blue vertical lines indicate the recommended age for vaccination (overlapping with red lines at birth for BCG and first polio vaccine), while the red lines indicate the outer ranges for the recommended age. The horizontal dotted lines represent coverage at end of follow-up. 2 The labels on the x-axis indicate the outer ranges for recommended vaccination age. One year of age is indicated as a scaling, but is also the upper recommended age for the measles vaccine. 3 Blue graphs line: Paarl; green graph line: Umlazi; red graph line: Rietvlei. [file 1471-2458-11-404-S3.TIFF]

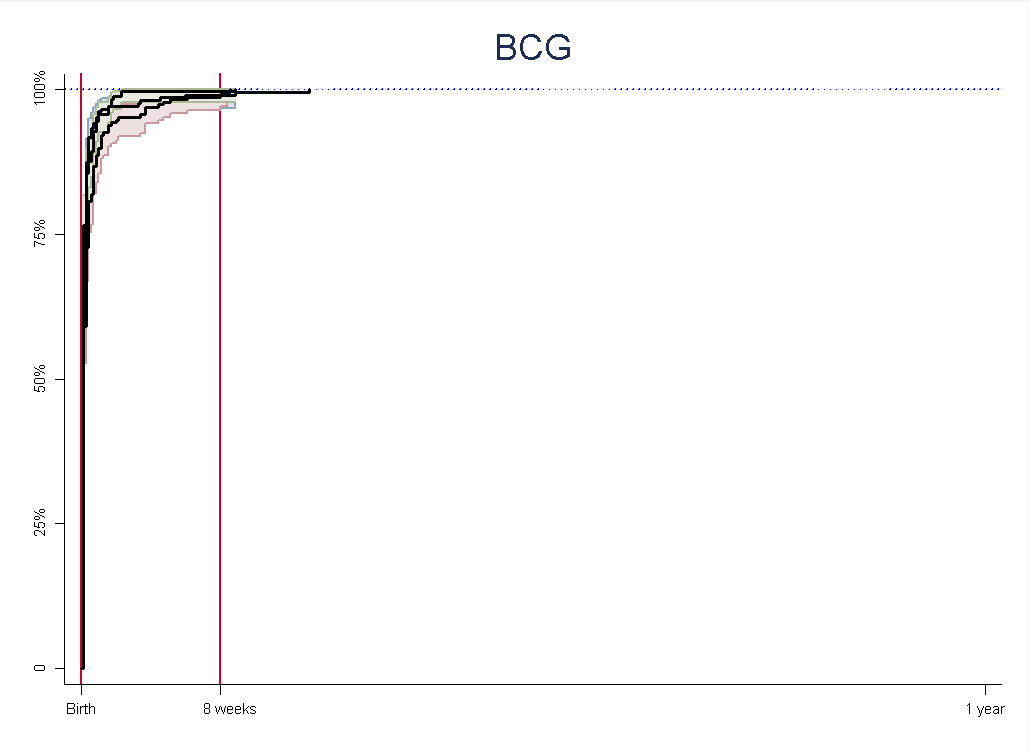

Supplement: Additional file 4 — Figure S4: Timing of the BCG vaccine given at birth for each site presented with Kaplan-Meier plots (inverse and cumulative). 1 The blue vertical lines indicate the recommended age for vaccination (overlapping with red lines at birth for BCG and first polio vaccine), while the red lines indicate the outer ranges for the recommended age. The horizontal dotted lines represent coverage at end of follow-up. 2 The labels on the x-axis indicate the outer ranges for recommended vaccination age. One year of age is indicated as a scaling, but is also the upper recommended age for the measles vaccine. 3 Blue graphs line: Paarl; green graph line: Umlazi; red graph line: Rietvlei. [file 1471-2458-11-404-S4.TIFF]

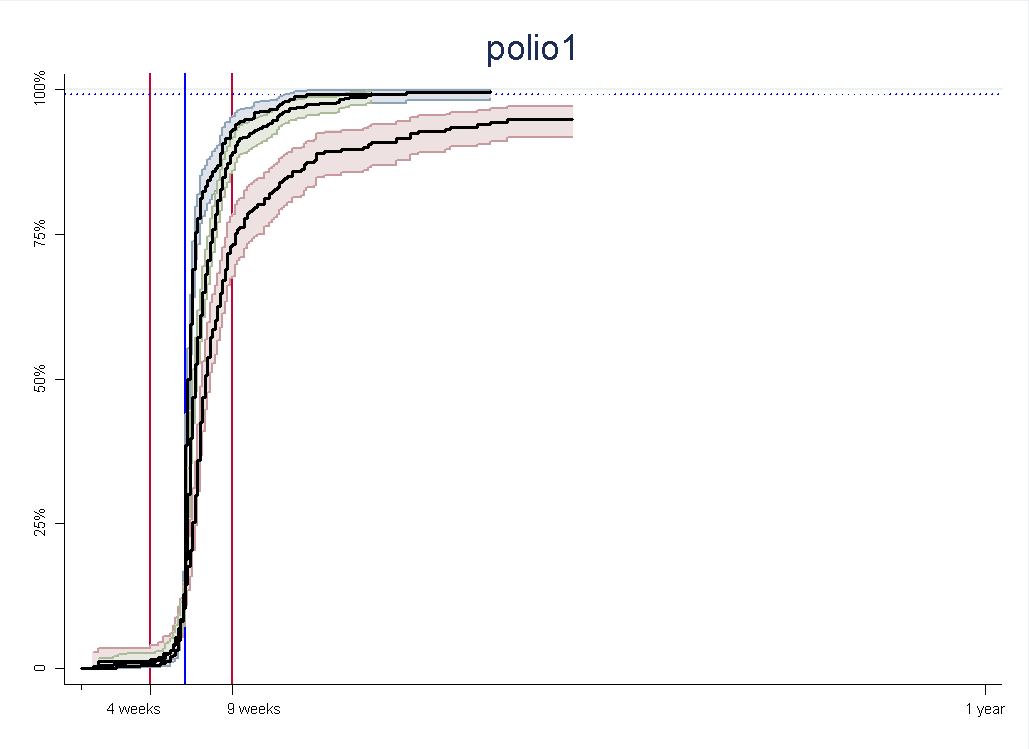

Supplement: Additional file 5 — Figure S5: Timing of the second oral polio vaccine (polio1) for each site presented with Kaplan-Meier plots (inverse and cumulative). 1 The blue vertical lines indicate the recommended age for vaccination (overlapping with red lines at birth for BCG and first polio vaccine), while the red lines indicate the outer ranges for the recommended age. The horizontal dotted lines represent coverage at end of follow-up. 2 The labels on the x-axis indicate the outer ranges for recommended vaccination age. One year of age is indicated as a scaling, but is also the upper recommended age for the measles vaccine. 3 Blue graphs line: Paarl; green graph line: Umlazi; red graph line: Rietvlei. [file 1471-2458-11-404-S5.TIFF]

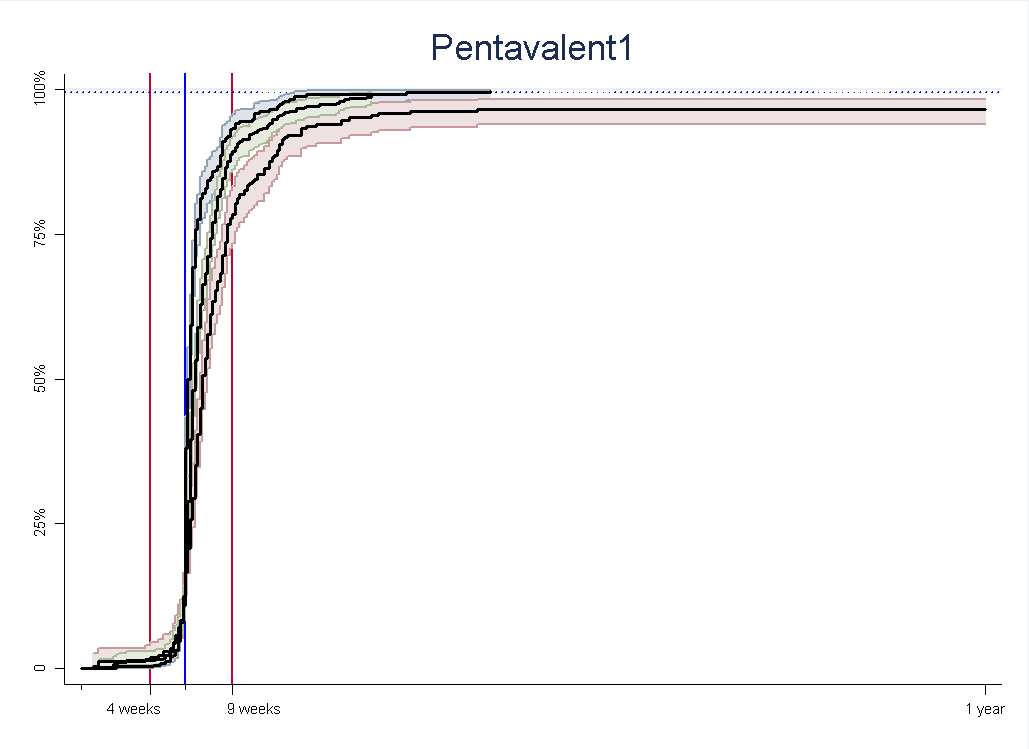

Supplement: Additional file 6 — Figure S6: Timing of the first pentavalent vaccine (pentavalent1) for each site presented with Kaplan-Meier plots (inverse and cumulative). 1 The blue vertical lines indicate the recommended age for vaccination (overlapping with red lines at birth for BCG and first polio vaccine), while the red lines indicate the outer ranges for the recommended age. The horizontal dotted lines represent coverage at end of follow-up. 2 The labels on the x-axis indicate the outer ranges for recommended vaccination age. One year of age is indicated as a scaling, but is also the upper recommended age for the measles vaccine. 3 Blue graphs line: Paarl; green graph line: Umlazi; red graph line: Rietvlei. [file 1471-2458-11-404-S6.TIFF]

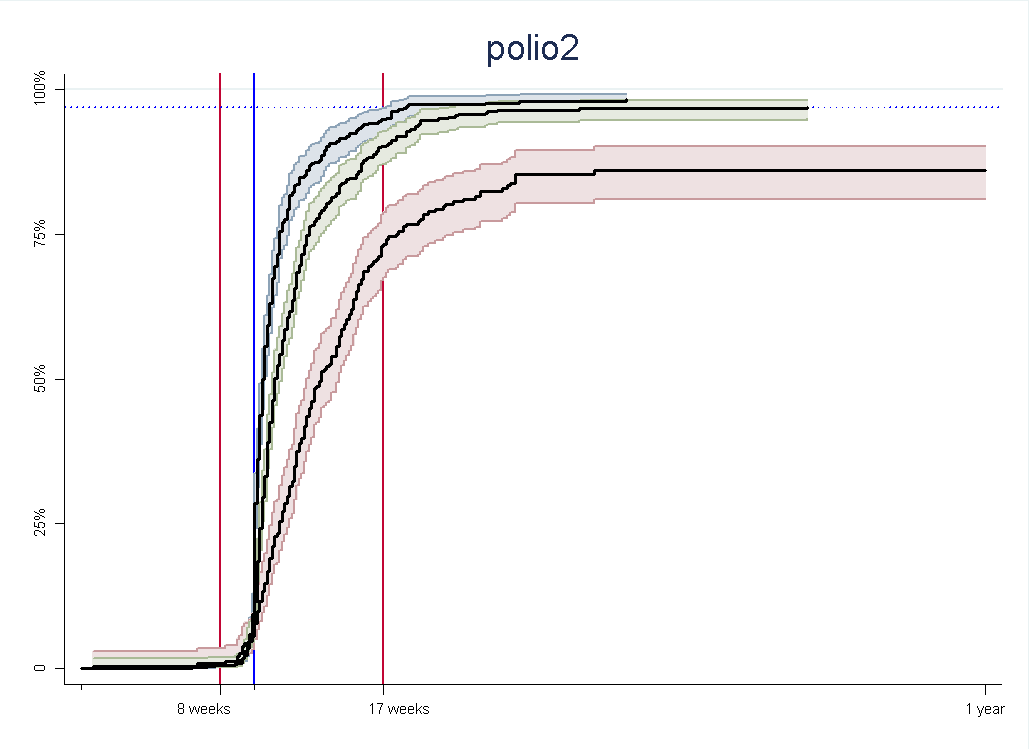

Supplement: Additional file 7 — Figure S7: Timing of the third oral polio vaccine (polio2) for each site presented with Kaplan-Meier plots (inverse and cumulative). 1 The blue vertical lines indicate the recommended age for vaccination (overlapping with red lines at birth for BCG and first polio vaccine), while the red lines indicate the outer ranges for the recommended age. The horizontal dotted lines represent coverage at end of follow-up. 2 The labels on the x-axis indicate the outer ranges for recommended vaccination age. One year of age is indicated as a scaling, but is also the upper recommended age for the measles vaccine. 3 Blue graphs line: Paarl; green graph line: Umlazi; red graph line: Rietvlei. [file 1471-2458-11-404-S7.TIFF]

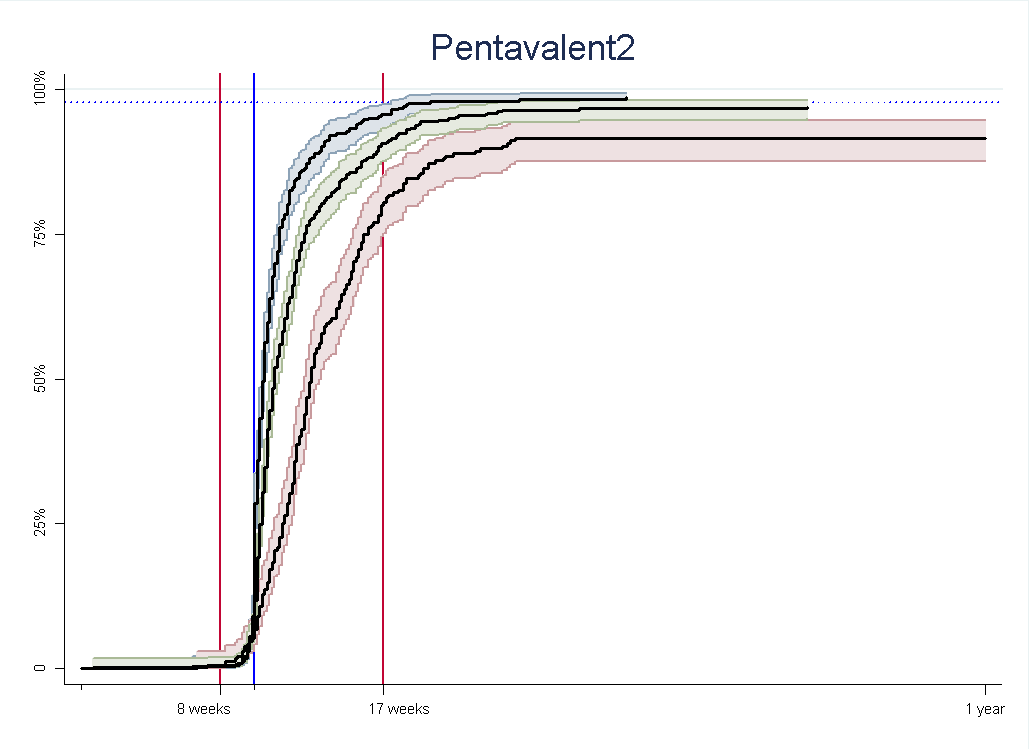

Supplement: Additional file 8 — Figure S8: Timing of the second pentavalent vaccine (pentavalent2) for each site presented with Kaplan-Meier plots (inverse and cumulative). 1 The blue vertical lines indicate the recommended age for vaccination (overlapping with red lines at birth for BCG and first polio vaccine), while the red lines indicate the outer ranges for the recommended age. The horizontal dotted lines represent coverage at end of follow-up. 2 The labels on the x-axis indicate the outer ranges for recommended vaccination age. One year of age is indicated as a scaling, but is also the upper recommended age for the measles vaccine. 3 Blue graphs line: Paarl; green graph line: Umlazi; red graph line: Rietvlei. [file 1471-2458-11-404-S8.TIFF]

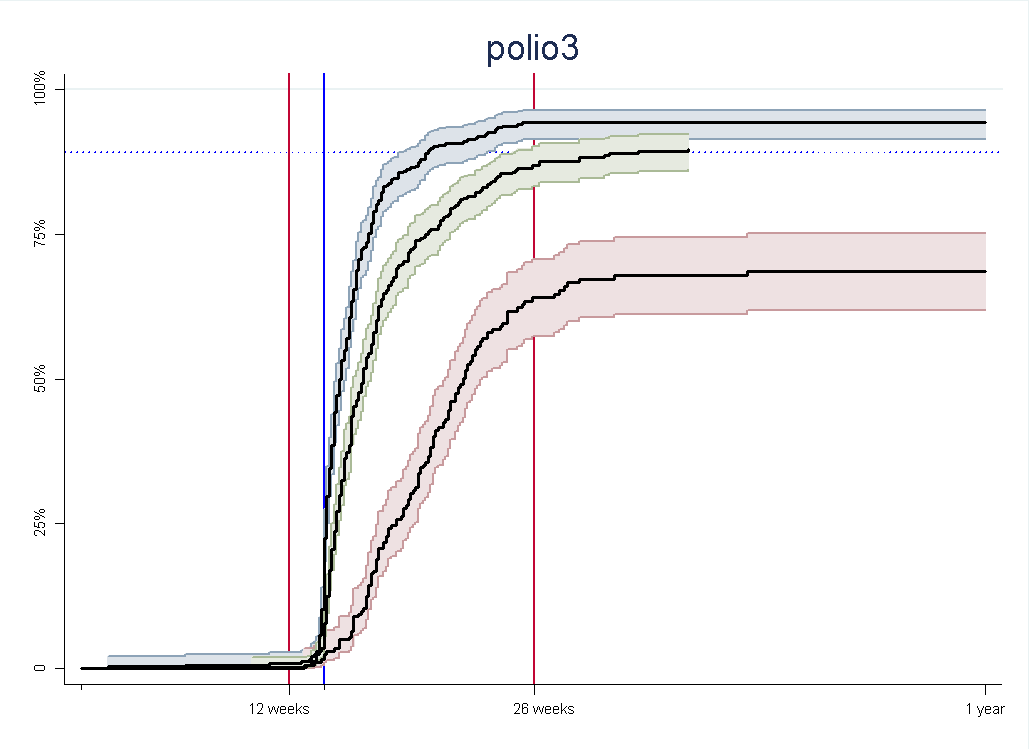

Supplement: Additional file 9 — Figure S9: Timing of the fourth oral polio vaccine (polio3) for each site presented with Kaplan-Meier plots (inverse and cumulative). 1 The blue vertical lines indicate the recommended age for vaccination (overlapping with red lines at birth for BCG and first polio vaccine), while the red lines indicate the outer ranges for the recommended age. The horizontal dotted lines represent coverage at end of follow-up. 2 The labels on the x-axis indicate the outer ranges for recommended vaccination age. One year of age is indicated as a scaling, but is also the upper recommended age for the measles vaccine. 3 Blue graphs line: Paarl; green graph line: Umlazi; red graph line: Rietvlei. [file 1471-2458-11-404-S9.TIFF]

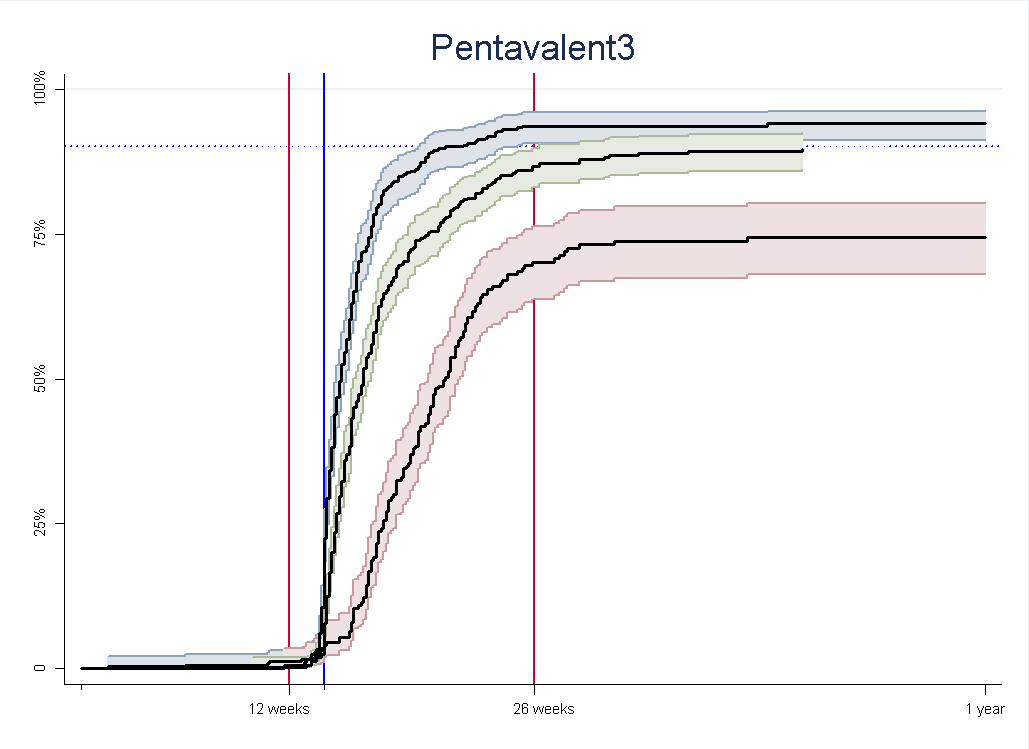

Supplement: Additional file 10 — Figure S10: Timing of the third pentavalent vaccine (pentavalent3) for each site presented with Kaplan-Meier plots (inverse and cumulative). 1 The blue vertical lines indicate the recommended age for vaccination (overlapping with red lines at birth for BCG and first polio vaccine), while the red lines indicate the outer ranges for the recommended age. The horizontal dotted lines represent coverage at end of follow-up. 2 The labels on the x-axis indicate the outer ranges for recommended vaccination age. One year of age is indicated as a scaling, but is also the upper recommended age for the measles vaccine. 3 Blue graphs line: Paarl; green graph line: Umlazi; red graph line: Rietvlei. [file 1471-2458-11-404-S10.TIFF]
